# Perceptions of patients undergoing percutaneous coronary intervention on pre‐operative education in China: A qualitative study

**DOI:** 10.1111/hex.13156

**Published:** 2020-11-13

**Authors:** Qiqi Zhuo, Hongmin Liang, Yangjuan Bai, Qiulan Hu, Ardani Latifah Hanum, Mingfang Yang, Yanjiao Wang, Wei Wei, Lan Ding, Fang Ma

**Affiliations:** ^1^ Department of Nursing The First Affiliated Hospital of Kunming Medical University Kunming China; ^2^ Cardiology Department The First Affiliated Hospital of Kunming Medical University Kunming China; ^3^ ICU in Geriatric Department The First Affiliated Hospital of Kunming Medical University Kunming China; ^4^ Urology Department The First Affiliated Hospital of Kunming Medical University Kunming China; ^5^ Psychiatric Department The First Affiliated Hospital of Kunming Medical University Kunming China; ^6^ Neurosurgery Department The First Affiliated Hospital of Kunming Medical University Kunming China; ^7^ General Surgery Department The First Affiliated Hospital of Kunming Medical University Kunming China

**Keywords:** China, percutaneous coronary intervention, pre‐operative health education, qualitative research

## Abstract

**Objectives:**

To explore the perceptions of patients undergoing percutaneous coronary intervention (PCI) regarding their pre‐operative health education.

**Methods:**

A qualitative study using semi‐structured, in‐depth interviews was conducted in one cardiology unit in China from July 2019 to December 2019. Purposeful sampling of 17 patients undergoing PCI was interviewed about their perceptions of pre‐operative health education. Thematic analysis of the transcribed data was then used to identify the themes.

**Results:**

Four themes emerged from the data:(a) triple roles of pre‐operative education with the categories of relief (reliving fear); burden (leading to stress); and meaningless (changing nothing); (b) family member involvement with the categories of shared responsibility and family members’ duty; (c) facilitators in the process of pre‐operative health education with the categories of emotional support, plain language and individualized pre‐operative education; (d) inhibitors in the process of pre‐operative health education with the categories of contradiction and threatening words.

**Conclusions:**

Pre‐operative health education for patients undergoing PCI should be aligned with the individual patients’ information‐seeking styles and personal differences, emphasizing individualized patient education. Traditional Chinese philosophy should be considered in the practice of pre‐operative education for patients undergoing PCI, which emphasizes family member involvement; at the same time, patient empowerment and self‐care should also be stressed. In addition, emotional support and plain language from health professionals are important in pre‐operative health education for patients undergoing PCI; contradiction should be avoided, and threatening words should be used with caution and with consideration for cultural variations during pre‐operative education for patients undergoing PCI.

## 1 BACKGROUND

As a treatment option for patients with coronary artery disease, percutaneous coronary intervention (PCI) is a common procedure for relieving obstruction in a stenotic coronary artery and is achieving better outcomes with the improvements in stent technology and the increased experience of practitioners.^1^, ^2^, ^3^, ^4^ It is reported that PCI has already experienced exponential growth, and its popularity is still expanding worldwide.^5^, ^6^ However, the rapid growth in PCI treatment has not been matched by the quality of care provided for patients undergoing PCI, which suggests quality improvement activities should be initiated to ensure that the care delivered to patients undergoing PCI is evidence‐based, safe and efficient.^7^ Typical problems encountered by patients undergoing PCI are psychological distress responses such as pre‐operative anxiety and depression, which may result in acute post‐operative hypertension and pain.^8^, ^9^ This has led to an increased focus on the reduction of pre‐operative psychological distress for patients undergoing PCI. It has been demonstrated that pre‐operative education can help decrease patients’ pre‐surgery anxiety and depression, improve patients’ compliance with pre‐operative requirements, alleviate their pain after surgery and benefit patients’ mental and physical health.^10^, ^11^, ^12^, ^13^ However, it has also been reported that, in some cases, pre‐operative education for cardiac surgery patients increased pre‐operation anxiety and did not benefit patient's recovery physically or psychologically.^9^, ^14^, ^15^ The conflicting results make the practice of pre‐operative education for patients undergoing PCI more confusing and complicated. Therefore, it is important to identify the perceptions of patients undergoing PCI on pre‐operative education so that health‐care professionals can understand their views and provide tailored care based on patients’ perceptions and preferences, which can increase the satisfaction of patients undergoing PCI and lead to better health outcomes.^16^

When exploring patients’ views about pre‐operative education, we should pay attention to the fact that patients view health treatments through the lens of their culture.^17^ Shiotani et al^18^ found that the cultural tradition of vegetarianism and fasting in India influenced peoples’ views about treatments for tuberculosis (such as recommendations to consume eggs and milk), which decreased adherence to tuberculosis treatments in rural India. It has been reported that cultural factors such as the breast being seen as less sexually attractive and a greater focus on safety instead of beauty in Chinese culture, as compared to American culture, influence women's views and choices regarding treatment for breast cancer. This resulted in the situation where a greater proportion Chinese‐American women with breast cancer preferred to have a radical mastectomy instead of breast‐conserving treatment, when compared with Latina, White or Black women with breast cancer.^19^, ^20^ Moreover, Dumit et al^21^ claimed that the strong reliance on God and acceptance of fate embedded in the Lebanese culture led many Lebanese people to believe that coronary interventions such as angioplasty were destined and ‘written’ by God. The above studies all suggest that people's culture is inextricably and meaningfully connected to their views about medical interventions and health needs, which highlights the fact that cultural factors should be taken into consideration in the exploration of the perceptions of patients undergoing PCI on pre‐operative education.

In Chinese culture, the heart is an organ resembling the monarch; wisdom and spirit stem from it. Therefore, receiving a diagnosis of heart disease signals a life‐threatening illness.^22^, ^23^ When thinking about treatments for heart disease, such as cardiac surgery or PCI, Chinese patients may become extremely scared and anxious at first due to the high risks involved with the treatment. However, they would later feel secure and calm because they believe that doctors will give them the best treatment, and trusting professionals matches the Confucian culture of Five Relationships (wulun, 五伦) and the core tenet of propriety (li,礼), which are deeply rooted in Chinese culture.^24^, ^25^ Five Relationships refer to the five dyadic relationships between ruler and minister, father and son, husband and wife, older and younger brother and friends, and the junior partner of the dyad owes strong duties of service and reverence to the senior partner.^26^ Respect and trust are usually given to experts such as doctors as stipulated by Five Relationships.^24^ Another factor contributing to patients’ calmness is their belief in fate and the adoption of a ‘do‐nothing’ approach, which result from the influences of Taoism and Confucianism and allow fate to take its course in the treatment of disease.^27^ Moreover, Chinese culture stresses the importance of family, and patients’ perceptions of treatments for heart disease involve their family members.^28^ Influenced by the long history and profound culture in China, the attitudes and views of Chinese patients undergoing PCI regarding their pre‐operative education might be unique and extraordinary. There is a scarcity of research concerning the perceptions of patients undergoing PCI regarding pre‐operative education in China. Therefore, the aim of this study was to examine the perceptions of Chinese patients undergoing PCI regarding their pre‐operative education, with the purpose of identifying information useful for developing a culturally appropriate pre‐operative education programme for patients undergoing PCI in China.

## 2 METHODS

### 2.1 Study design

A descriptive phenomenology study was adopted to understand the lived experiences and views about pre‐operative education of patients undergoing PCI; this emphasizes the description of personal experiences and aims to disclose and explore the meaning of those experiences.^29^, ^30^, ^31^ Face‐to‐face in‐depth interviews with patients who had undergone PCI were conducted to obtain qualitative data, allowing the exploration of individuals’ rich and detailed experiences in their own words.^28^

### 2.2 Setting and sample

This study was undertaken in one cardiology department of a tertiary referral hospital in Yunnan Province, China. Yunnan is located in a border region of China, and many ethnic minority groups live together there, which might provide cultural‐specific information to help understand the perceptions of patients undergoing PCI regarding pre‐operative education. The purposive sampling method is a frequently applied, conceptually driven approach in qualitative research, which can supply the researcher with rich and fruitful information to explore in answering the research question.^32^ Therefore, we used a purposive sampling method with the strategy of homogenous sampling for this study. Selecting participants who have experienced the phenomena of interest is essential to obtaining detailed and meaningful experiential data.^33^ Only patients who have experienced pre‐operative education for PCI may provide the necessary detailed information. To make sure that we could obtain accurate and rich information, participants were required to meet the following inclusion criteria: (a) having undergone a PCI electively during this hospitalization; (b) being in a stable state without psychiatric problems; (c) being able to communicate well with the interviewer; and (d) being recommended by their charge nurses as talkative and outspoken. These criteria affected the contribution the potential participants could provide to the study and were important for guiding the selection process.^32^ The reason for relying on charge nurses to pre‐select ‘talkative and outspoken’ participants is that they spend more time with the hospitalized patient than any other clinician group,^34^ which means that charge nurses might know patients’ personalities better than other health‐care providers, and they can help pre‐select the appropriate participants. Sampling continued concurrently with data analysis until the saturation point was reached at the 14th interview, and no new themes were identified from the subsequent interviews. In order to ensure that no new information would appear, a further three participants were interviewed.

### 2.3 Data collection

Before the interview, demographic data such as admission diagnosis, type of procedure, gender, age, nationality and education level were collected using a self‐made questionnaire. Interview data were collected using semi‐structured in‐depth interviews between July 2019 and December 2019. Patients receiving PCI are normally discharged within 48‐72 hours after the operation in the hospital, which is consistent with other research findings.^35^ We consider the day after the operation as the appropriate time for the interview, because patients are in a relatively stable state and not in a rush to leave the hospital at this time, making them more available for the interview on that day. All interviews were conducted privately in the ward when the patients were alone. Each interview was audio‐taped, and important information such as movements and facial expressions was recorded in notes. The average duration for each interview was approximately 27 minutes (with a range of 20‐39 minutes). The interview guide was developed based on the pilot interviews. Participants were asked to recall their pre‐operative education experiences, and the following topics guided the interviews: (a) How do you feel about the pre‐operative education you received for your PCI? (b) What factors facilitate and encumber the effect of pre‐operative education for patients receiving PCI? The interviews were conducted by a female graduate nursing student (ZQQ) who had completed the required qualitative research courses and was previously unknown to the participants. During the interviews, the interviewer gave the potential participants a letter with comprehensive information about the study and explained to them the purpose of the study at the beginning. Then, the potential participants were given time to think before giving their written informed consent. No one rejected the request for participation or dropped out of the study. The reviewer tried to hide her opinions during data collection and reviewed her bias of the research before the conduct of the interviews. Only the 14th participant was interviewed for a second time, due to being interrupted by treatment during the original interview. The interviews, original transcriptions and data analysis were in Chinese. Each recording and memo was transcribed verbatim into Chinese within 24 hours by the interviewer. Participants were asked to verify the accuracy of the information discussed during the interview before the end of the interview; hence, the interview transcripts were not returned to participants for comment. After data analysis, the selected quotations were translated from Chinese into English by two bilingual translators to ensure that meaning was retained.

### 2.4 Data analysis

All transcribed interviews were subjected to thematic analysis, and the transcripts were imported into the Nvivo 11 software package for easy coding and retrieval to identify the emerging themes.^36^ In order to obtain a comprehensive understanding of the participant's perspectives and experiences and to identify meaningful themes, the process of analysis included repeated reading and rereading of the transcribed texts in order to become familiarized with the data; initial codes were then identified with the use of line‐by‐line analysis. Similar codes were aggregated together and sorted into themes, which were reviewed by connecting their relationships. To eliminate the risk of bias, the interviewer (ZQQ) and corresponding author (MF) with expertise in qualitative data analysed the data independently. Findings were discussed thoroughly by the research team, and agreement was reached through discussion where differences in analysis appeared. Since the participants had been discharged from the hospital when the analysis was completed, we did not get feedback on the findings from the participants.

### 2.5 Ethical considerations

The study was approved by the ethics committee of the hospital. Participants were given written information about the study's aims and procedures and about their right to withdraw at any time. Written informed consent was obtained from each participant. Participants’ names were replaced with numerical codes, and the digital audio recordings were only used for this study, which protects the privacy of participants and maintains the confidentiality of data.

## 3 FINDINGS

Seventeen participants aged from 46 to 75 were interviewed in one‐on‐one in‐depth interviews. The majority of the participants were of Han ethnicity (fourteen) and three belonged to minority groups. This is due to the fact that some ethnic minority patients could not communicate well with the interviewer because they could only speak their local dialect, which resulted in their exclusion from the study. The majority of participants in this study were male (15 men and 2 women) and, therefore, the sample does not equally represent both genders. However, prior studies have shown that the prevalence of coronary heart disease in men is higher than that in women, and female patients are less likely to receive primary PCI with acute coronary syndrome compared to male patients in China.^37^, ^38^ This might explain why the proportion of males in the study was much higher than that of females. The demographic characteristics of the participants can be seen in Table 1.

**Table 1:** Participant demographics

Characteristics	Participants, N (%)
Gender	
Male	15 (88.2)
Female	2 (11.8)
Age group, y	
40‐49	1 (5.9)
50‐59	8 (47.1)
60‐69	5 (29.4)
70‐79	3 (17.6)
Ethnicity	
Han	14 (82.3)
Naxi	1 (5.9)
Yi	1 (5.9)
Jinpo	1 (5.9)
Education	
Primary school	3 (17.6)
Junior high school	8 (47.1)
High school	2 (11.8)
Bachelor's degree	4 (23.5)
Type of PCI	
Coronary angiogram	1 (5.9)
Angiogram and stent	14 (82.3)
Angiogram, stent and angioplasty	2 (11.8)
Admission diagnosis	
Coronary heart disease	9 (52.9)
Chest pain	2 (11.8)
Chest distress	4 (23.5)
Heart failure	1 (5.9)
Tachycardia	1 (5.9)

The following four major themes emerged from the findings: (a) triple roles of pre‐operative education; (b) family member involvement; (c) facilitators in the process of pre‐operative education; and (d) inhibitors in the process of pre‐operative education.

### 3.1 Theme 1: Triple roles of pre‐operative education

When talking about their feelings regarding patient education, participants expressed their different experiences and triple roles of pre‐operative education emerged, which included three categories: (a) relief; (b) burden; and (c) meaningless. Participants considered pre‐operative education could relieve their anxiety, but they also felt burdened with the information given during the education, and some participants did not care about that.

### 3.2 Category 1: Relief

Most participants indicated that they were scared and anxious before PCI, which meant facing an unknown situation. The unpredictable situation represented a huge health threat to them because it involved the important organ‐heart. Most participants thought that pre‐operative education helped relieve their fear and anxiety by providing relevant information such as the surgical procedure, what they should do before the surgery, the recovery process and so on:

*‘Before the operation, I was frightened and nervous, because I knew nothing about the surgery. Would I die or be disabled after the surgery? Is it a high risk? What should I do now? Lots of questions surrounded me, which troubled me and even made me sleepless. Fortunately, the pre‐operative education helped me a lot. The nurse came and told me lots of information about the surgery and what I should do before the operation. For my part, the knowledge about PCI lessened the uncertainty and it was a real relief for me’*.

Participant 9

### 3.3 Category 2: Burden

However, some participants mentioned pre‐operative education had become the source of fear and depression. They could not bear the fear that came from the information given during the pre‐operative education. The more they knew about PCI, the more they were afraid of it:

*‘In the pre‐operative education process before the surgery, the nurse told me lots of information about PCI and something we should do before and after the surgery. Since then, I couldn’t stop thinking about the risk of PCI; is it safe? Would I do as I was told before and after surgery…all the time. I felt stressed and nervous and I couldn’t sleep well. That was a burden for me’*.

Participant 14

### 3.4 Category 3: Meaningless

Some participants proposed that they did not care about pre‐operative education. They believed that their lives were determined by fate, and doctors could make them well. Whether they were given pre‐operative education or not was meaningless:

*‘I don`t care if the medical staff give me pre‐operative education or not*.

*They are experts to be trusted and our destiny is fated. I appreciated the education they gave me, but I don’t think this information can change anything and I was a passive receiver of the education, which didn’t make any sense’*.

Participant 6

### 3.5 Theme 2: Family member involvement

Participants mentioned that family members should take care of them during hospitalization, and pre‐operative education should involve family members. There were two categories: (a) shared responsibility and (b) family member's duty.

### 3.6 Category 1: Shared responsibility

Some participants stated that they were not only sick but also faced with various psychological pressures. They needed help from other people, and family members should share the responsibility during the PCI treatment process, including pre‐operative education:

*‘I felt exhausted and tired…I couldn’t totally understand what they were talking about in the pre‐operative education, but my wife was beside me. With help from my wife, I knew what PCI was and what I should do before and after the surgery. Her involvement was very important’*.

Participant 4

### 3.7 Category 2: Family members’ duty

Some participants indicated that since they were patients, they should depend on their family members in every aspect. Family members should take care of them and arrange everything for them. Pre‐operative education should be conducted to their family members only, and it had nothing to do with them:

*‘It's more appropriate for the physician to talk to my family members about the surgery, right? As a 60 year old person, I let my children listen to the pre‐operative education. That is their responsibility and what I should do is to rest’*.

Participant 2

### 3.8 Theme 3: Facilitators in the process of pre‐operative education

Participants reflected that there existed some factors which facilitated the effect of pre‐operative education. The qualitative data resulted in three categories pertaining to factors facilitating the process of pre‐operative education: (a) emotional support; (b) plain language; and (c) individualized pre‐operative education.

### 3.9 Category 1: Emotional support

Most participants stated that before PCI, they were concerned about their illness, afraid of surgery and worried about their recovery. During the pre‐operative education process, these negative emotions were often ignored by health‐care professionals, and emotional support could facilitate the effect of pre‐operative education:

*‘The pre‐operative education I received was at a high standard. But I was nervous during my hospital stay because the surgery concerned the heart, which frightened me all the time. The terrible emotional stress hindered my understanding of the information. I really hope that the medical staff could have given me emotional support, which would have relaxed me a lot and the effect of the education might have been better’*.

Participant 10

### 3.10 Category 2: Plain language

All participants mentioned that medicine was too complicated to understand. They could not fully understand the content of the pre‐operative education, especially some technical words and terminology. Therefore, it is very important to explain complex medical phenomena using plain language:

*‘I was particularly impressed by my experience of pre‐operative education. It was conducted by a young doctor who spent five minutes talking to me. He said that the heart was like a house with four rooms, and the coronary arteries are the electric wiring for the house. Normally, the doors of different rooms in the house would be opened and closed regularly to control the supply of blood to your body. Now the wires were blocked and the doors couldn’t work. A stent should be put into the wire to make it work…’*

Participant 6

### 3.11 Category 3: Individualized pre‐operative education

Most participants stated that different patients had different backgrounds and that, accordingly, their needs might be different. If the health professionals could give pre‐operative education according to their personal characteristics and requirements, the effects would be much better:

*‘I believe that pre‐operative education should be based on the patients’*

*educational level, social background, personality, emotional situation, etc Anyway, our needs are different…some people want to learn more, while some people want to learn less’*.

Participant 11

### 3.12 Theme 4: Inhibitors in the process of pre‐operative education

Participants talked about the factors hampering the practice of pre‐operative education. The qualitative data resulted in two categories pertaining to inhibitors in the process of pre‐operative education: (a) contradiction and (b) threatening words.

### 3.13 Category 1: Contradiction

A few participants reflected that sometimes different medical staff gave different opinions during pre‐operative education, which led to confusion about whom to believe. The inconsistency served to reduce the trust of patients towards medical staff and caused confusion and stress:

*‘There are many medical staff working in the ward. Before the operation, different medical staff talked to me, but what they told me was contradictory. I was confused and at a loss. Who is right and what should I do?’*

Participant 6

### 3.14 Category 2: Threatening words

Some participants stated that they were scared of the words about risk information, such as ‘you`re going to die if you don`t do this’, ‘you`re seriously ill’. This kind of threatening words increased patients’ psychological burden and is not conducive to the physical and mental health of the patient:

*‘During pre‐operative education, the doctor told me that I would die within no more than one year if I didn’t receive PCI He talked a lot about the serious complications which were over‐exaggerated, and I was frightened… I didn’t want to hear anything’*.

Participant 12

## 4 DISCUSSION

There are mixed results regarding the role of pre‐operative education, which can make health‐care professionals confused about whether pre‐operative education facilitates or hinders patients’ well‐being. The results of the current study indicate that pre‐operative education for patients undergoing PCI is complicated from the patients’ point of view and is not simply a facilitator or an inhibitor to patient well‐being. This is something that requires further exploration and investigation.

The data reveal that pre‐operative education was a relief for most patients undergoing PCI, which has been reported in other research findings and highlights the importance of pre‐operative education in improving the physical and psychological well‐being of patients undergoing PCI.^11^, ^39^ Our study demonstrated that pre‐operative education was a burden for some patients undergoing PCI, which might be explained by the ‘Blunting Hypothesis’ proposed by Miller et al According to this theory, individuals are categorized into two different coping styles on the basis of how they deal with threat‐related cues: monitors (information seekers) or blunters (information avoiders).^40^ When threatened with a threatening situation, such as PCI (a life‐threatening procedure), monitors benefit from large amounts of information, while blunters benefit from avoiding it because it constitutes a source of stress.^41^, ^42^ Hence, it was not surprising that patients with information avoiding style felt pre‐operative education with a lot of information was a burden for them. These results further highlight the need to give education which is aligned to patients’ different coping styles.

Our study suggests that some patients undergoing PCI considered pre‐operative education to be meaningless, which might be related to the Chinese philosophy that influences individuals’ ways of living and thinking about health. Confucianism, Taoism and Buddhism are the core ideas of traditional Chinese philosophy.^43^ Buddhism emphasizes fate as one of the factors that determines health.^44^ Therefore, many Chinese people believe in fate and are content to simply let fate take its course. Furthermore, they perceive death as natural and an extension of life,^22^, ^44^ which might lead to the belief that pre‐operative education before PCI is useless, because what they suffer and whether they will recover are up to fate. Another reason might be that Chinese patients have a high degree of respect for health professionals, and they tend to play a subordinate role in encounters between doctors and patients.^28^, ^44^, ^45^ This might lead them to believe that there is no necessity for pre‐operative education, since health professionals are experts and are able to treat their health problem. Probyn et al^46^ also reported that patients undergoing PCI had faith in the medical professionals to ‘fix’ them and saw PCI treatment as their only option, which resulted in their reluctance to participate in decision‐making discussions and learn about the procedure. Health professionals should be aware of this phenomenon and help patients to engage more actively in their health‐care strategies for better patient empowerment, which can in turn improve their health outcomes.

In our analysis, some patients undergoing PCI emphasized the shared responsibility between patients and their family members in the pre‐operative education process, which might be explained by the importance of family in Chinese culture. Respect for parents and loyalty to family are the main teachings of Confucianism,^44^ which leads Chinese families to feel strongly obliged to look after one another, while caring for a sick person is considered a family duty.^28^, ^43^, ^45^, ^47^ Therefore, family members of patients undergoing PCI should be involved in the pre‐operative education process and patient education should be more family‐centred, which is also evidenced by other research.^48^ Some patients considered that the pre‐operative education was their family members’ duty. This might be explained by the Chinese traditional culture of family members overprotecting the patient, such as family members doing everything for the patient to keep them from being tired, which results in patients’ heavy reliance on their family members in the treatment of disease.^22^, ^28^, ^49^ This traditional culture is in conflict with self‐care recommendations for patients undergoing PCI, which are important for adults with coronary artery disease.^50^ Hence, health‐care professionals should encourage patients undergoing PCI to be involved in pre‐operative education for better self‐care to maintain their health and well‐being.^51^

Emotional support was cited as an important facilitator in pre‐operative education for most patients undergoing PCI, which is also reported by Higgins et al^39^ It has been shown that emotional support, including empathy, encouragement, warm greetings, eye contact, listening attentively and leaving time for the patient, can help establish rapport between health professionals and patients, which contributes to the success of health education.^52^ Plain language was another facilitator for the pre‐operative education of all patients undergoing PCI, which has been demonstrated in other research. Mentrup^53^ found in his qualitative synthesis study that participants wanted health information to be provided in an easy‐to‐understand language rather than complex medical terms. Svavarsdottir et al^54^ suggested that patients undergoing PCI prefer health education be delivered in lay language. In our study, individualized pre‐operative education based on different backgrounds, personalities and needs facilitated the patient education outcomes. It has been reported that individualized pre‐operative education brings positive surgical experience to patients and nurses, decreases patient anxiety and stress and reduces post‐operative pain levels,^10^, ^11^, ^40^, ^54^, ^55^, ^56^ highlighting the fact that pre‐operative education should be tailored to help patients fare better psychologically, behaviourally and physiologically.

As mentioned by a few participants, contradiction encumbered the practice of pre‐operative education. It has been reported that the confusing and conflicting information from different professionals in pre‐operative education made patients anxious and lost, which resulted in non‐adherence to the requirements before PCI and the failure of the education.^53^, ^57^ As evidenced by this study, threatening words is shown to be another obstacle to the delivery of pre‐operative education. These words about risk information might frighten cardiac patients and contribute to their depression and anxiety.^53^ However, Svavarsdóttir`s research indicates that patients hope the educator will conduct patient education in a strict and harsh way.^54^ This is possibly because health professionals and patients understand ‘risk’ in different ways; health professionals typically approach risk analytically by focusing on evidence, while patients may perceive risk based on personal experience and associational meanings.^58^ There is not any satisfactorily ‘correct’ way to talk about risk information in patient education, and health professionals face a series of dilemmas in their communications with coronary heart disease patients, calling for a willingness to hear the patient narrative and consider cultural difference.^53^ Confucianism stresses the maintenance of harmony and the threatening words might induce conflict between health professionals and patients.^43^ To maintain harmony, patients want to be spoken with in non‐threatening words to avoid conflict with others, which reminds us once again that cultural variations, and personal differences should be considered during pre‐operative education.

### 4.1 Strengths and limitations

The perceptions of patients undergoing PCI regarding pre‐operative education in the Chinese context are rarely explored in the literature. This study provides a unique insight into patients’ views about per‐operative education, highlighting culture as a key factor in influencing people's attitudes and views about intervention, which brings new and interesting information for health professionals to develop health education programmes that can benefit patients. One of the main limitations of the study was that the participants were from Yunnan Province only, which reduces generalizability. As the sample consisted predominantly of people of Han ethnicity, some unique ethnic cultural influences on patients’ perspectives on pre‐operative education may be neglected. Participants were selected with the criterion of being talkative and open‐minded, which leads towards an indirect exclusion of some anxious or depressed patients who might not be talkative, so we can not guarantee that all voices were sufficiently heard. Further studies can be conducted in many other hospitals and among different ethnic groups to enrich the findings, and experiments can be carried out to investigate the results of pre‐operative education based on patients’ perspectives, which can facilitate the exploration of the role of pre‐education more clearly in the current mixed research results.

## 5 CONCLUSIONS

The perceptions of patients undergoing PCI regarding pre‐operative education in one hospital in China show the complexity and challenges of providing pre‐operative education for patients undergoing PCI to help ensure improved outcomes of the success of the operation. More is not always better; pre‐operative education should consider information‐seeking styles, individual background and personal needs, in order to promote the effect of education, which highlights that individualized pre‐operative education is key to the practice of pre‐operative education. It is without any doubt that family members should be encouraged to participate in the pre‐operative education of patients undergoing PCI to support them in Chinese culture, but patients should not totally depend on their caregivers and self‐care is also important. Furthermore, patient empowerment should be enhanced to make them engage more actively in their health care and pre‐operative education as well. We should focus not only on what information is given but also on how such information is delivered and received in the education process. The findings call for emotional support and plain language from health professionals regarding their facilitating roles in the pre‐operative education of patients undergoing PCI. In addition, contradictions should be avoided and threatening words should be used with caution, taking account of cultural variations during pre‐operative education for patients undergoing PCI. Although the study was conducted in one hospital within one specific region in China, the preliminary findings illuminate issues related to ways for improving the effect of pre‐operative education for patients undergoing PCI in China.

## CONFLICT OF INTEREST

None.

## AUTHOR CONTRIBUTIONS

Qiqi Zhuo (First author) acquired and interpreted the data and drafted the manuscript. Hongmin Liang and Yangjuan Bai analysed and interpreted the data. Qiulan Hu has made substantial contributions to conception and design. Ardani Latifah Hanum revised the manuscript. Mingfang Yang and Yanjiao Wang acquired of data. Wei Wei has made substantial contributions to conception and design. Lan Ding analysed and interpreted the data. Fang Ma (Correspondence author) involved in analysis and interpretation of data, revising the manuscript and given final approval of the version to be published.

## ETHICAL APPROVAL

This study was approved by the Ethics Committee of the First Affiliated Hospital of Kunming Medical University.

## Data Availability

The data that support the findings of this study are available on request from the corresponding author. The data are not publicly available due to privacy or ethical restrictions.
